# Bleeding assessment in a large cohort of patients with Osteogenesis Imperfecta

**DOI:** 10.1186/s13023-024-03054-8

**Published:** 2024-02-12

**Authors:** Koert Gooijer, Gabriëla Heidsieck, Arjan Harsevoort, Daniëlle Bout, Guus Janus, Anton Franken

**Affiliations:** 1 Expert Center for Adults with Osteogenesis Imperfecta, Isala Hospital, Dokter van Heesweg 2, PO Box 10400, 8025 AB Zwolle, The Netherlands; 2 Department of Clinical Genetics, Amsterdam Reproduction and Development Research Institute, Amsterdam University Medical Centers, University of Amsterdam, Amsterdam, The Netherlands

**Keywords:** Osteogenesis Imperfecta, Bleeding disorders, Haemorrhage, Hematoma, Vessel fragility, Self-BAT

## Abstract

**Background:**

Osteogenesis Imperfecta (OI) is characterised by bone fragility. Among several features, easy bruising and multiple case reports on haemorrhagic events have been reported. This paper describes the diverse manifestations of bleeding and bruising in a large cohort of 328 OI patients. The aim of this study is to provide insight in the diverse aspects and therapeutic considerations of bleedings in OI.

**Methods:**

This descriptive cohort study was conducted at the National Expert Center for adults with OI in the Netherlands. Bleeding was assessed by the validated self-bleeding assessment tool (Self-BAT) The tool was distributed among 328 adults with different clinically confirmed types of OI.

**Results:**

195 of 328 invited patients (completion rate 60%) with OI type 1 (n = 144), OI type 3 (n = 17) and OI type 4 (n = 34), aged between 18 and 82 years, completed the tool. Self-BAT scores were above the normal range in 42% of all patients. For males Self-BAT scores were increased in 37% with a mean score of 3.7, ranged between 0 and 18. For females the Self-BAT scores were increased in 44% with a mean of 5.4 and a range of 0–24. No statistical differences in OI subtypes were found.

**Conclusions:**

Bleeding tendency appears to be a relevant complication in OI patients as this study confirms the presumption of bleeding tendency. There are specific recommendations to clinicians who treat OI patients to consider an assessment of bleeding tendency and use potential interventions to reduce haemorrhagic complications and improve quality of life.

**Supplementary Information:**

The online version contains supplementary material available at 10.1186/s13023-024-03054-8.

## Background

Osteogenesis Imperfecta (OI), commonly defined as ‘brittle bones’ disease, is pathogenetically based on an hereditary collagen type I synthesis disorder, most often due to an autosomal dominant mutation in COL1A1 or COL1A2 genes. In addition to collagen type I genes, OI can be caused by multiple proteins connected to different parts of collagen biosynthesis [1]. Therefore it includes broader characteristics like blue sclerae, hearing loss, dental problems, ligamentous laxity and short stature [2]. The degree of impaired production of collagen type 1 is based on dominant pathogenic gene variants and results in a heterogeneous clinical expression historically classified in 5 types [3]. The prevalence of OI is about 6–7 per 100,000 [4]. Easy bruising and bleeding are prominent features of some heritable connective tissue disorders such as Ehlers-Danlos, and is also commonly reported by OI patients [5–7]. The literature for OI in relation with bleeding disorders is sparse and often dated [8–11]. Bleeding disorders in heritable disorders of the connective tissue can be a result of fragility of capillaries and perivascular connective tissue, but can also be caused by a clotting problem due to platelet dysfunction, a defect in fibrinolysis or vascular components of the haemostasis [6]. Although diagnosis of severe bleeding disorders such as moderate or severe hemophilia can be well defined, the distinction between individuals with or without a mild bleeding disorder is often difficult. The use of a bleeding assessment tool (BAT) in mild bleeding disorders can be more distinctive than a complete laboratory workup [12–14].

Since clinical appreciation of presence and severity of bleeding symptoms is a fundamental step in the evaluation of a possible bleeding disorder in OI, the aim of this study is to describe the diverse aspects of bleeding and bruising in our whole cohort of OI patients using a structured questionnaire. We also intend to provide insight in the clinical consequences and give therapeutic considerations of bleeding in OI due to surgery, tooth extraction, menstruation and obstetrics.

## Methods

A nationwide descriptive cohort study was undertaken in the National Expert Center for adults with OI, Isala Hospital, Zwolle, the Netherlands. All known patients in our clinic with a clinical diagnosis of OI according to the OI classification of van Dijk and Sillence [3] were invited to fill in the self-bleeding assessment tool (Self-BAT) between October 2019 and August 2020. The Medical Ethics Committee of the Isala Hospital, Zwolle, The Netherlands, confirmed that the Medical Research Involving Human Subjects Act does not apply (reference number: 190513). All patients who were invited for this study signed an informed-consent form for study participation.

### Evaluation of bleeding tendency

The Self-BAT is a self-administered bleeding assessment tool validated in Canada and the Netherlands [15, 16] and is based on the International Society on Thrombosis and Haemostasis Bleeding Assessment Tool (ISTH-BAT) [17, 18]. The 14 Self-BAT domains cover epistaxis, cutaneous bleeding, minor wounds, haematuria, gastrointestinal bleeds, oral cavity bleeds, prolonged bleeding after trauma, surgeries or tooth extraction, menorrhagia, postpartum haemorrhage, muscle, joint and central nervous system bleeds and other bleedings. Each domain scores from 0 (absence of bleeding symptoms) to 4 (symptoms requiring extensive medical intervention). The distinction between 0 points and 1 point is of critical importance since score 1 means the symptom meets the minimal criteria defining a significant bleeding. This distinction between the different scores is described by the ISTH Scientific and Standardization Committee [19] and independently selected by 2 trained researchers (KG and HB). Different scores were reassessed and resolved by consensus. A total bleeding score is calculated by sum of scores for all BAT domains. A total bleeding score in men ≥ 4 and in women ≥ 6 was defined as an increased bleeding tendency [15, 16].

### Data collection

All data obtained from the Self-BAT were collected digitally and analysed using IBM SPSS Statistics Version 24. Variables were presented as numbers (n) and frequencies (%), mean and SD and median and interquartile range (IQR). Comparison of means between OI types 1, 3 and 4 was done using ANOVA with Bonferroni comparison for post-hoc testing. *P* values ≤ 0.05 were considered to indicate statistical significance.

## Results

Of 328 invited OI patients, 225 patients (69%) returned the questionnaire. In total, 30 patients did not fully complete the questionnaire and were therefore excluded from the analysis. Therefore 195 questionnaires were available for analysis (completion rate 60%). No reasons for non-completion or signs of selective response were found.

### Participant characteristics

The study population was predominantly of Dutch origin with a male/female ratio of 71/124. It concerned individuals with OI type 1 (n = 144), type 3 (n = 17) and type 4 (n = 34) (Table 1), with diverse genetic causes of OI. The mean age of this population at time of inclusion was 43,7 years (SD 15.6), with a median of 40 and ranged between 18 and 82 years (Table 1). Three patients (1.5%) were known with von Willebrand disease (Additional file 1: Table S2). No other bleeding disorders were reported. Surgery and dental extractions were relatively common, 91% (n = 177) of the patients had undergone surgery and 86.2% (n = 168) had experienced a dental extraction. Of all 124 women, 91.9% (n = 114) ever had a menstrual period and 42.7% (n = 53) had been pregnant. Women with OI type 3 had never been pregnant. Of the women who had been pregnant, 47 had at least 1 delivery. The reason why 6 of the pregnant women did not give birth was not known.

**Table 1:** General descriptive data study population

		OI type 1	OI type 3	OI type 4
		N = 144	N = 17	N = 34
*Sex, N (%)*	Male	49 (25)	7 (4)	15 (8)
	Female	95 (49)	10 (5)	19 (10)
*Age in years, mean (SD)*		44.7 (15.2)	31.7 (13.1)	45.4 (16.3)

### Bleeding assessment

#### Self-BAT score

**Fig. 1:**
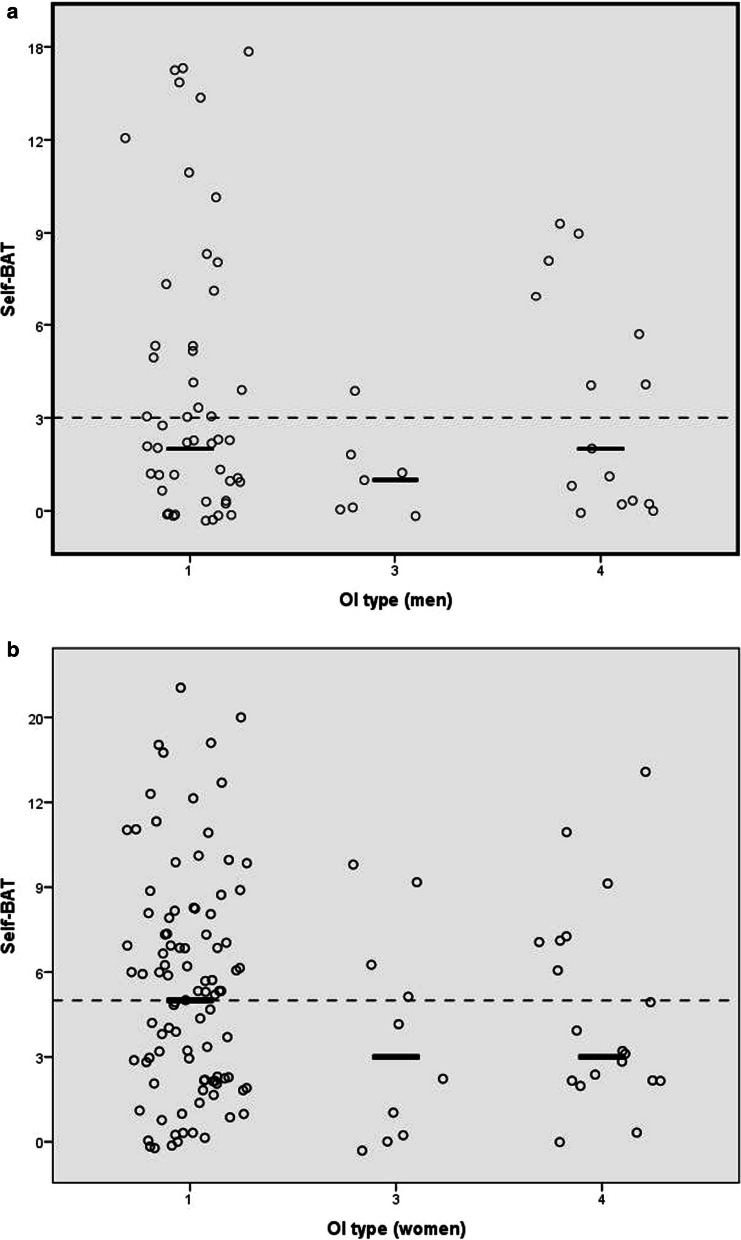
Correlation between Self-BAT bleeding score and OI types. Black lines are medians. Dashed line is cut-off score for men and women

Self-BAT scores were above the normal range in 42% of all patients. For males Self-BAT scores were increased in 37% (n = 26) with a mean score of 3.7, ranged between 0 and 18. For females the Self-BAT scores were increased in 44% (n = 55) with a mean of 5.4 and a range of 0–24. There was no statistically significant difference between the OI types regarding severity of each bleeding symptom or total Self-BAT scores (Fig. 1). The correlation between age and Self-BAT score is negligible (R^2^ = 0.012).

**Fig. 2:**
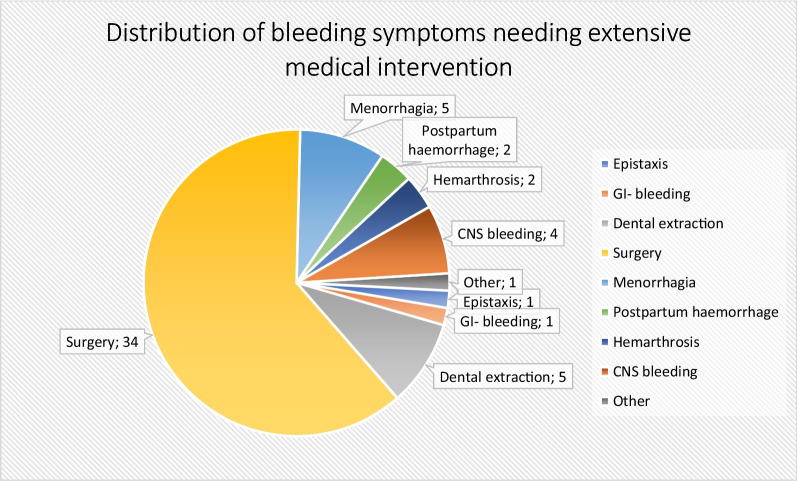
Distribution of bleeding symptoms needing extensive medical intervention

Extensive medical intervention (a domain score of 4) was needed in 23% of the patients (n = 45) and mostly needed in the domains of surgery (n = 34), menorrhagia (n = 5) and dental extraction (n = 5). (Fig. 2) 5% Of the patients (n = 10) required extensive medical intervention on more than 1 Self-BAT domain. The number of patients with significant bleeding per different bleeding domains is shown in Table 2. The specification of severity of bleeding symptoms (score 1–4) in the different domains are shown in Additional file 1: Table S1. A symptom was scored as “present” if the patient indicated this in the questionnaire and scored as “significant” if the patient had a score of 1 or more on the Self-BAT for this item. Overall, the most common significant symptoms were menorrhagia (59.7% of all women) and cutaneous bleeding (50.8%). Other prevalent symptoms were postpartum hemorrhage (34%), bleeding from minor wounds (32.8%) and bleeding after surgery (30.3%). The least common symptoms were haematuria (2.1%) and cerebral bleeding (2.6%) (Table 2).

**Table 2:** Analysis of the Self-BAT scores in 195 patients with OI

*Self-BAT domains*	Present*	Significant bleeding	Significant bleeding leading to increased total Self-BAT, n (%)
	n (%)	n (%)	
*Cutanous bleeding*	171 (87.7)	99 (50.8)	58 (58.6)
*Bleeding from minor wounds*	184 (94.4)	64 (32.8)	45 (70.3)
*Menstruation (of 124 woman women)*	114/124 (91.9)	74 (59.7)	45 (60.8)
*Surgery*	177** (90.8)	59 (30.3)	52 (88.1)
*Dental extraction*	168** (86.2)	40 (20.5)	31 (77.5)
*Epistaxis*	145 (74.4)	39 (20)	26 (66.7)
*Muscle hematomas*	32 (16.4)	32 (16.4)	22 (68.8)
*Hemarthrosis*	18 (9.2)	18 (9.2)	15 (83.3)
*Other bleeding*	17 (8.7)	17 (8.7)	11 (64.7)
*Childbirth (of 124 woman)*	47/124** (37.9)	16 (34)	10 (21.3)
*Oral cavity bleeding*	36 (18.5)	9 (4.6)	7 (77.8)
*Gastro intestinal bleeding*	14 (7.2)	6 (3.1)	4 (66.7)
*Central nervous system bleeding*	5 (2.6)	5 (2.6)	5 (100)
*Haematuria*	24 (12.3)	4 (2.1)	3 (75)

*“Present” corresponds to the number of patients who experienced a cutaneous, minor wound, nose, muscle, joint, oral cavity or urinary tract bleeding regardless of significance of bleeding

**Corresponds to the number of patients who experienced a dental extraction, surgery, women who menstruated and women who had at least 1 delivery, with or without bleeding

## Discussion

Bleeding problems are often reported by patients with OI and in case reports, but large cohort studies reporting on bleeding tendency of adult patients are not performed. Clinical appreciation of the presence and severity of bleeding symptoms is a fundamental step in the evaluation of a possible bleeding disorder in OI and therefore this study investigated bleeding tendency in a large cohort of adult OI patients using a structured BAT. In 42% of the included OI patients the Self-BAT score was increased compared to the normal range [20].

### Bleeding after surgery

Surgery is frequently needed in our population. In this cohort 91% of all patients underwent surgery. Because 17% of all included patients with OI reported that they required blood transfusion after surgery, awareness of this bleeding risk is of critical importance. Identification of risk factors in OI patients before surgery can result in less bleeding incidents and reduce the need for transfusion because attention to mild bleeding symptoms can ameliorate bleeding diathesis when drugs, such as desmopressin and antifibrinolytics, are used when necessary [14, 21–23]. There are several studies describing excessive bleeding due to surgery in OI patients, despite normal pre-operative haematological assessment. Wong et al. reported bleeding complications despite normal preoperative coagulation studies in 30% of the study participants (7 out of 23) and Langness and Behnke et al. in 11% of the study participants (9 out of 80) [24, 25]. A recent study of Rothschild et al. reported significant intraoperative blood loss in 17% of 205 surgeries among 83 OI patients without link to coagulation disorders [26]. Also Morton et al. (n = 1), Wood et al. (n = 2), Waters et al. (n = 1) and Mondal (n = 1) describe bleeding diathesis without explanatory coagulation disorders [27–30]. Several studies try to discover a link between bleeding diathesis and laboratory abnormalities, but all were inconclusive [5, 11, 31–34]. The use of a structured bleeding questionnaire as is used in this study seems to be far more useful than laboratory measurements at this time, because correlation between levels of a specific factor and the severity of bleeding symptoms is still usually poor. This may be because standard tests poorly reflect in vivo haemostasis. The contribution of many factors (e.g. vessel fragility or fibrinolysis) cannot be measured. Also a genomic search for the molecular basis of inherited clotting and platelet defects may not be as useful as a good questionnaire because often variability in penetrance and expressivity [35], coinheritance of haemostatic defects or superimposed genetic modifiers make the relationship between genotype and phenotype less stringent than previously appreciated [12, 14]. For a mild bleeding disorder as OI a clinically driven “bleeding” diagnosis based on anamnestic risk factors can be of more benefit than preoperative laboratory investigation. Obaji et al. applied desmopressin and/or tranexamic acid to a group of patients with a significant bleeding history with no reproducible abnormality with the standard tests of haemostasis, and found no bleeding in 90% of patients undergoing an intervention [36].

### Post extraction bleeding (PEB)

21% Of the patients in this study experienced unusual bleeding due to dental extraction. PEB (Post extraction bleeding) has divergent definitions but is a well-recognized, frequently encountered complication in dental practice with an varying incidence between 0 and 26% [37]. Post extraction bleeding has been attributed to various factors that can be broadly classified as local and systemic. Locally soft tissue or bone bleeding can occur due to traumatic extraction leading to laceration of blood vessels. Also inflammation at the site of extraction, traumatic extraction, and failure of the patient to follow post‐extraction instructions have also been associated with PEB. Systemic factors include platelet problems, coagulation disorders or excessive fibrinolysis, and inherited or acquired problems (medication induced) [38]. In the current study we did not differentiate the underlying factors that can cause bleeding and a literature review did not reveal any previous reports for bleeding risk after tooth extraction in relation to patients with OI. However, further differentiation in cause of post extraction bleeding would be very useful since patients with OI have also often dentinogenesis imperfecta [39] and are at risk of bisphosphonate related osteonecrosis of the jaw [40].

### Heavy menstrual bleeding (HMB)

In our cohort 60% of the women had significant menorrhagia. In 40% of all patients who menstruated, abnormal menstruation contributed to an increased total BAT score. This means that menstrual bleeding disorder in OI contributes significantly to an increased BAT score. Menorrhagia is the most frequent health problem for a woman during reproductive life, occurs in about 30% of women, is underdiagnosed and poorly treated [41]. It has a major impact on a woman’s quality of life for which the NICE guideline on heavy menstrual bleeding encourages clinicians to focus their interventions on improving quality of life rather than focusing on blood loss [42]. Besides this already large problem in the general population, menorrhagia is a prominent feature of most bleeding disorders [43–45]. Among patients with moderate to severe Von Willebrand Disease in the Netherlands a prevalence of menorrhagia of nearly 80% was demonstrated [46]. No literature is available for the prevalence of menorrhagia in patients with OI, but our study suggests an increased prevalence of menorrhagia in OI compared to the general population. With regard to the major impact on quality of life and the relatively large prevalence of HMB in OI it should be noted that a substantial proportion of women with menorrhagia with abnormal laboratory haemostasis has shown to respond to therapy with desmopressin and/or tranexamic acid with decreased bleeding and improved quality of life [47]. Therefore, pro-active treatment for HMB in OI should be considered.

### Postpartum haemorrhage (PPH)

In our cohort 53 female patients stated they had ever been pregnant. Of these, 47 patients reported ever having given birth. 16 Out of 47 women (34%) experienced significant PPH which is much higher than the incidence of PPH in many high-income countries [48]. Recent research on pregnancies with OI shows significant higher rates of blood transfusion compared with US normative data, a significant increase in pre-pregnancy bleeding and a non-significant increase in post-partum bleeding compared with non-OI pregnancies [49, 50]. PPH though is worldwide known as a major contributor to maternal death [51] and even mild haemostatic abnormalities can be a significant risk factor for severe PPH [52]. Also previous PPH results in a much higher risk of recurrent PPH during a subsequent delivery [53, 54]. The high prevalence of PPH in OI as found in this study highlights the need for awareness of possible bleeding tendency in pregnant woman with OI. Veen et al. found that PPH can be the first symptom of an inherited bleeding disorder, but also emphasises assessing the bleeding history as 75% of the women with a bleeding disorder had additional bleeding symptoms other than PPH [55]. In our cohort the percentage of women with PPH and an elevated BAT score, meaning at least 1 significant bleeding symptom other than PPH, was 63%. The use of a structured questionnaire such as the ISTH-BAT can also be of benefit in pregnant woman with OI, especially since administration of tranexamic acid to women with post-partum haemorrhage reduces deaths due to bleeding by nearly one third, with no evidence of any adverse effects or complications [56].

### Strengths and limitations

We carried out the largest nationwide study ever performed in adult patients with OI focused on bleeding assessment. With this study a lot of valuable information was obtained which gave a unique insight in the features of bleeding tendency in OI. The results of this study provide a substantial contribution to our knowledge on the symptoms of bleeding disorders in patients with OI. In addition, increased awareness of bleeding complications among health care providers involved in the care of patients with OI may contribute to improved quality of life in these patients.

Albeit the Self-BAT is a validated questionnaire for different mild bleeding disorders [15, 16], it is not specially developed for OI. Due to the explorative design of this cohort study and the absence of a matched control group, no relative risk estimates could be calculated. Also the use of an self-reporting BAT has some limitations because symptoms reported by patients may be influenced by the fact that several patients are already familiar with the potential relation between OI and an increased bleeding tendence. Furthermore, recall of symptoms and treatments can be difficult, especially when the presence of bleeding symptoms spans a lifetime and might be sporadic due to a low amount of haemostatic challenges. This is also claimed by Moenen et al., who also emphasises that although an elevated BAT score increases the patients likelihood of having an mild bleeding disorder, a specific disorder cannot be diagnosed based on the BAT [57, 58]. The main value of the BAT is well established in the assessment of a bleeding disorder and provides a structured, complete diagnostic anamneses. Abnormalities can justify extensive laboratory testing because a distinctive bleeding history is a prerequisite for the diagnosis of any bleeding disorder [12, 59]. We used the BAT for an descriptive purpose to gain more insight in the bleeding tendency in OI. For this reason we also described the different domains of the Self-BAT, which has been done earlier [60].

## Conclusions

The abundant manifestations of bleeding and bruising described in this large cohort suggests that a mild bleeding disorder should be considered in patients with OI. Despite some limitations of a self-report questionnaire, there are very likely more bleeding problems in OI compared to the normal population. Since bleedings due to surgery, tooth extraction, menstruation and obstetrics are more common and can have major clinical consequences, proactive therapy might be considered in patients with a high bleeding tendency detected on the BAT. Many OI patients underwent multiple invasive procedures because of fractures and dentinogenesis imperfecta, possibly with a undiscovered history of bleeding. This means that patients are at risk of experiencing unnecessary and potentially debilitating haemorrhagic symptoms if a bleeding tendency is not identified. The use of a structured bleeding questionnaire as is used in this study seems to be far more useful than laboratory measurements at this time. We recommend clinicians who treat OI patients to assess bleeding tendency with the use of a Bleeding Assessment Tool. We also recommend consideration and evaluation of potential interventions to reduce haemorrhagic symptoms with the use of desmopressin and/or tranexamic acid which might be sufficient to reduce haemorrhagic symptoms and (in menorrhagia) improve quality of life. These interventions had as described in literature no evidence of adverse effects, while patients might be prevented from being exposed to potential risks associated with the administration of blood products. Future studies will be required to further define the bleeding phenotype in OI and to investigate a possible correlation with genotype. Also OI specific studies into results of preventive medication on bleeding tendency and validation of reliability and feasibility of the Self-BAT in OI are important issues for future research.

### Supplementary Information

**Additional file 1**. **Supplemental table 1.** Number of patients with bleeding symptoms for each Self-BAT domain including scores that contributed to increased Self-BAT scores. **Supplemental table 2.** Scores of the patients known with Von Willebrand disease.

## Data Availability

All datasets generated during and/or analyzed during the current study are not openly available due to reasons of sensitivity and are available from the corresponding author upon reasonable request. Data are located in controlled access data storage at Isala Hospital.
