# Supplementary material for: Bleeding assessment in a large cohort of patients with Osteogenesis Imperfecta
Source: Orphanet J Rare Dis. 2024 Feb 12;19:61. doi: 10.1186/s13023-024-03054-8 (PMC10860322; doi:10.1186/s13023-024-03054-8)
Supplement: Supplementary file 1 — Additional file 1. Supplemental table 1. Number of patients with bleeding symptoms for each Self-BAT domain including scores that contributed to increased Self-BAT scores. Supplemental table 2. Scores of the patients known with Von Willebrand disease. [file 13023_2024_3054_MOESM1_ESM.docx]

Supplemental table 1. Number of patients with bleeding symptoms for each Self-BAT domain including scores that contributed to increased Self-BAT scores.

|  | 1 point: n, (%) | patients with high BS: n; (%) | 2 points: n, (%) | patients with high BS: n; (%) | 3 point: n, (%) | patients with high BS: n; (%) | 4 point: n, (%) | patients with high BS: n; (%) |
| --- | --- | --- | --- | --- | --- | --- | --- | --- |
| Epistaxis | 19 (9,7) | 10 of 19 | 8 (4,1) | 7 of 8 | 11 (5,6) | 8 of 11 | 1 (0,5) | 1 of 1 |
| Cutanous bleeding | 53 (27,2) | 21 of 53 | 28 (14,4) | 19 of 28 | 18 (9,2) | 18 of 18 | 0 | 0 |
| Bleeding from minor wounds | 48 (24,6) | 30 of 48 | 11 (5,6) | 11 of 11 | 4 (2,1) | 4 of 4 | 0 | 0 |
| Haematuria | 2 (1) | 1 of 2 | 1 (0,5) | 1 of 1 | 1 (0,5) | 1 of 1 | 0 | 0 |
| Gastro intestinal bleeding | 4 (2,1) | 2 of 4 | 0 | 0 | 1 (0,5) | 1 of 1 | 1 (0,5) | 1 of 1 |
| Oral cavity bleeding | 5 (2,6) | 4 of 5 | 2 (1) | 2 of 2 | 2 (1) | 1 of 2 | 0 | 0 |
| Dental extraction | 18 (9,2) | 11 of 18 | 4 (2,1) | 2 of 4 | 13 (6,7) | 13 of 13 | 5 (2,6) | 5 of 5 |
| Surgery | 9 (4,6) | 4 of 9 | 2 (1) | 2 of 2 | 14 (7,2) | 14 of 14 | 34 (17,4) | 32 of 34 |
| Menorrhagia [n=124] | 24 | 12 of 24 | 43 (22) | 18 of 43 | 2 (1) | 1 of 2 | 5 (2,6) | 4 of 5 |
| Postpartum haemorrhage [n=47] | 4 | 2 of 4 | 3 (1,5) | 3 of 3 | 7 (3,6) | 3 of 7 | 2 (1) | 2 of 2 |
| Muscle hematomas | 17 (8,7) | 11 of 17 | 7 (3,6) | 4 of 7 | 8 (4,1) | 7 of 8 | 0 | 0 |
| Hemarthrosis | 9 (4,6) | 7 of 9 | 2 (1) | 1 of 2 | 5 (2,6) | 5 of 5 | 2 (1) | 2 of 2 |
| Central nervous system | 0 (0) | 0 | 0 | 0 | 1 (0,5) | 1 of 1 | 4 (2,1) | 4 of 4 |
| Other bleeding | 10 (5,1) | 6 of 10 | 6 (3,1) | 5 of 6 | 0 | 0 | 1 (0,5) | 1 of 1 |

Supplemental table 2. Scores of the patients known with Von Willebrand disease

| Von Willebrand disease | Patiënt A | Patiënt B | Patiënt C |
| --- | --- | --- | --- |
| Epistaxis |  | 2 | 4 |
| Cutanous bleeding | 1 | 3 | 3 |
| Bleeding from minor wounds |  | 2 | 1 |
| Oral cavity bleeding |  |  | 3 |
| Dental extraction |  | 4 | 4 |
| Surgery | 4 | 4 | 1 |
| Menstruation | 1 |  | 2 |
| Central nervous system bleeding | 3 |  |  |
| Total | 9 | 15 | 18 |
